# Fully Biodegradable Composites: Thermal, Flammability, Moisture Absorption and Mechanical Properties of Natural Fibre-Reinforced Composites with Nano-Hydroxyapatite

**DOI:** 10.3390/ma12071145

**Published:** 2019-04-08

**Authors:** Pooria Khalili, Xiaoling LIU, Zirui ZHAO, Brina Blinzler

**Affiliations:** 1 Ningbo Nottingham New Materials Research Institute, University of Nottingham Ningbo China (UNNC), Ningbo 315100, China; pooria.khalili@gmail.com (P.K.); zy18541@nottingham.edu.cn (Z.Z.); 2 Department of Industrial and Materials Science, Chalmers University of Technology, 412 96 Gothenburg, Sweden; brina.blinzler@chalmers.se

**Keywords:** poly(lactic acid) (PLA), natural fibre (NF), nano-hydroxyapatite (nHA), flammability, mechanical properties

## Abstract

Natural fibre-reinforced poly(lactic acid) (PLA) laminates were prepared by a conventional film stacking method from PLA films and natural fabrics with a cross ply layup of [0/90/0/90/0/90], followed by hot compression. Natural fibre (NF) nano-hydroxyapatite (nHA) filled composites were produced by the same manufacturing technique with matrix films that had varying concentrations of nHA in the PLA. Their flammability, thermal, moisture absorption and mechanical properties were analysed in terms of the amount of nHA. The flame behavior of neat PLA and composites evaluated by the UL-94 test demonstrated that only the composite containing the highest quantity of nHA (i.e., 40 wt% nHA in matrix) was found to achieve an FH-1 rating and exhibited no recorded burn rate, whereas other composites obtained only an FH-3. The thermal degradation temperature and mass residue were also observed, via thermogravimetric analysis, to increase when increasing concentrations of nHA were added to the NF composite. The tensile strength, tensile modulus and flexural modulus of the neat resin were found to increase significantly with the introduction of flax fibre. Conversely, moisture absorption was found to increase and mechanical properties to decrease with both the presence of NF and increasing concentrations of nHA, and subsequent mechanical properties experienced an obvious reduction.

## 1. Introduction

Polymeric thermosets such as vinyl ester, unsaturated polyester and epoxy are chosen for most composites due to their resistance to chemicals, hydrophobicity and suitable mechanical performances. However, thermoplastic matrices have recently gained significant attention in production industries such as construction, automotive and packaging. Bio-based polymers such as thermoplastic starch, poly(lactic acid) (PLA) and poly(3-hydroxybutyrate) (PHB) can be substituted for polyethylene, polystyrene or polypropylene. Petroleum based polymers can be replaced with some bio-based counterparts to deal with disposal issues and problems of diminishing fossil fuel stocks. For many applications where mechanical recycling is an issue, biodegradable polymers are of great interest, as they can be made to be biodegradable and compostable [1]. PLA, amongst other bio-based polymers, possesses relatively high crystallinity, melting point and stiffness, which demonstrates a greater commercial potential [2,3]. However, for engineering applications, in order to obtain the required mechanical performance, this type of polymer must be reinforced.

Recent regulations on the recyclability of materials and environmental requirements compel manufacturers and research institutions to develop composites from renewable sources. Plant fibres (PF) i.e., flax, ramie, jute, hemp and sisal are biodegradable, renewable and economical to use, have high specific modulus and strength, and have low density [4,5,6]. PFs possess approximately 40% lower density than glass fibres, which leads to the manufacturing of lighter components than polymeric parts reinforced with glass fibres [7,8]. This is crucial for applications related to transportation resulting in reduced emissions and enhanced fuel efficiency [9]. Therefore, the eco-advantages and the reduced mass of these materials make them competitive with synthetic fibre-reinforced composites. However, biocomposites are considered flammable when compared with the traditional fibre-reinforced plastics e.g., carbon and glass fibre composites. Therefore, bio-based composites are more flammable than the traditional composites. This limits the usage of biocomposites in applications where the fire regulations are stringent, for instance the aviation and railway industries [10]. The enhancement of fire and thermal resistivity of biocomposites (e.g., PLA-based biocomposites) is required even for less stringent industries like automotive and packaging.

Inorganic fillers such as silica nanoparticles at high concentration were used to impart high thermal stability to the cellulose-based composites, as obtained from thermogravimetric analysis (TGA) [11]. Nano-clay was also used in a different study to improve the thermal stability and flame retardancy of natural fibre polymer composites [12]. In another investigation, the incorporation of halloysite nanotubes into natural fibre polymeric composites was reported [13]. The thermal degradation temperature was observed to enhance with the incorporation of these nano-fillers. Except for the nano-inorganic additives mentioned, the thermal properties and flame retardancy of natural fibre-reinforced plastics has been improved by the inclusion of various flame-retardants (FRs) into polymers. For instance, the introduction of 3–7 wt% of expandable graphite (EG) [10] and 5–15 wt% ammonium polyphosphate (APP) [6] into 20 wt% natural fibre-reinforced polymer provided 0 s drip flame time from a vertical Bunsen burner test, reduced gross heat of combustion as obtained from bomb calorimeter and increased the mass residue from thermogravimetric analysis. However, the addition of these fillers was observed to reduce the mechanical performance of the FR-filled composites. This is because high concentrations of inorganic additive, especially compared to organic fillers, are necessary to impart fire resistivity into composites [14]. The high concentrations lead to the embrittlement and deterioration of the mechanical performance of composites.

Nano hydroxyapatite (nHA), ${\mathrm{Ca}}_{10}{\left({\mathrm{PO}}_{4}\right)}_{6}{\left(\mathrm{OH}\right)}_{2}$, is an inorganic bio-filler that has medical applications and is the main calcium phosphate phase present in bone. nHA does not present high strength, which makes it unfit for load-bearing applications [15]. Akindoyo et al. [15] showed that addition of 10 wt% nHA into PLA enhanced the content of mass residue from 0.35% to 6.17% at 750 °C from thermogravimetric analysis. This demonstrates higher thermal performance of nHA than PLA as inclusion of nHA can result in enhanced char residue.

To the best of the authors’ knowledge, few literature studies have reported the improved thermal performance of PLA with the addition of nHA [5,16], and no investigation has highlighted the enhanced flame resistivity and char formation of natural fibre PLA laminates with the inclusion of nHA particles. The water absorption and mechanical behavior of natural fibre-reinforced PLA composites containing nHA additives have also not been reported thus far. The composites were made of fully green materials i.e., bio-based polymer, natural fibre and bio-filler to produce an environmentally friendly composite. In this research, 100% biodegradable composites were developed using various loadings of nHA and these nano-composites were compared with neat PLA and flax fibre-reinforced laminate. The aim of this study was to utilize the good thermal properties of nHA to develop flax fabric PLA composites with improved flame retardancy and thermal resistivity. The formulations produced were investigated based on flammability, thermal, mechanical and water absorption behaviors.

## 2. Materials and Methods

### 2.1. Materials

Unidirectional flax fabrics were purchased from Easy Composites Ltd (Stoke-on-Trent UK) and had the mass over area and density of 150 g/m^2^ and 1.5 g/m^3^, respectively. Nano-hydroxyapatite (nHA), Ca_10_(PO_4_)_6_(OH)_2_ ≥ 99.5%, was supplied by Yunduan New Materials, Weifang, China. The particle size was less than 40 nm, had the density of 3.16 g/cm^3^ and had pH of 7.41. The mass loss after drying was 0.59% and mass loss after burning was 2.59%. The poly (lactic acid) (PLA, Ingeo^TM^ bio-based polymer PLA3251D) was provided by NatureWorks LLC (Minnetonka, MN, USA) and had the specific gravity of 1.24 g/cm^3^.

### 2.2. Processing

The nanocomposites were compounded using an internal mixer. The PLA along with nHA was compounded at 190 °C for 20 min at a mixing speed of 200 rpm. The quantity of nHA was prepared at 20 wt%, 30 wt% and 40 wt% in PLA matrix. The compounding materials (PLA/nHA) and pure PLA were pressed using a hot press machine to produce films to be used as the matrix. The films with a thickness of roughly 0.3 mm were produced using an XLB50 ( flat vulcanization press (YueQing TOPS machinery CO., Ltd, Shanghai, China) at a pressure of 3 MPa for the period of 10 min at the temperature of 190 °C. The PLA and PLA/nHA films were cold pressed for 2 min, immediately after removal from the hot press. It is worth noting that the PLA, nHA and films were kept in a convectional laboratory oven for 24 h at 50 °C before any stage of processing and the flax fabrics were kept in an oven at 80 °C prior to the hot compression.

Composites were produced by a conventional film-stacking method, which is constructed by adding alternating layers of matrix film and plant fabric. The cross-ply of [0/90/0/90/0/90] was utilized to produce the laminates. The prepared films and flax fabric were hot pressed into 280 × 160 × 2 mm^3^ plates using the same hydraulic hot press set at a pressure of 5 bar for 10 min at 190 °C for consolidation, and subsequently the composite plates were cold pressed to room temperature for 2 min. The prepared composites were named CPLA (control), CN20, CN30 and CN40. CPLA was made of flax fabric and PLA, and the number in front of CN20, CN30 and CN40 (nano-composites) denotes the amount of nHA in the matrix. The CPLA and nano-composites contained 30% fibre volume fraction. Neat PLA was also fabricated for comparison purposes.

### 2.3. Flammability Test

The flame behaviours of the PLA, control and composite laminates were examined using the UL-94 test in accordance with ISO 1210 [17]. The samples were placed horizontally and the burn rate of each sample in the horizontal orientation was reported. The sample is classified as FH-1 if the combustion front does not pass the 25 mm mark. It is categorized as FH-2 if the combustion front passes the 25 mm mark, but does not pass the 100 mm mark. The burnt length is added to the classification designation. It is classified as FH-3 if combustion front passes the 100 mm mark and the rate of burning does not exceed 75 mm/min for samples measuring a thickness < 3 mm. If the rate of burning surpasses the mentioned value, it is categorized as FH-4. If the flame does not continue after the initial mark, which is 25 mm from the end of specimen, the burn rate is denoted “not applicable” (NA).

### 2.4. Thermogravimetric Analysis (TGA)

Thermogravimetric analysis (TGA) was conducted on a TA instrument (SDT Q600) (New Castle, DE, USA, 2016) to investigate the thermal stability of the composites. The samples of approximately 20 mg were placed in a platinum crucible and were heated from 30 °C to 600 °C in a nitrogen environment. The flow rate and ramping rate were set to 50 ml/min and 20 °C/min, respectively. The corresponding TGA and differential thermal gravimetry (DTG) were obtained

### 2.5. Differential Scanning Calorimeter (DSC) Test

The differential scanning calorimeter (DSC) test was conducted on a TA machine. (SDT Q20) (New Castle, DE, USA, 2016). Samples weighing 17–19 mg were heated from room temperature (25 °C) to 200 °C at a ramping rate of 10 °C/min with a nitrogen flow rate of 50 ml/min. The same heating rate and flow rate were used to cool down the samples to the room temperature. The crystallinity of PLA based matrix was obtained using the Equation (1) [18] below:

(1) $$X_{\mathrm{DSC}}\%=\frac{\Delta H_{m}-\Delta H_{c}}{\Delta H_{m}^{{}^{\circ}}}\times \frac{100}{w}$$

where ΔH_m_ is the melting enthalpy, ΔH_c_ is the enthalpy of cold crystallization, $\Delta H_{m}^{{}^{\circ}}$ is 93.7 J/g for pure crystalline PLA and w is the mass fraction of the PLA-based matrix.

### 2.6. Scanning Electron Microscopy (SEM)

Scanning electron microscopy (SEM, ZEISS Sigma/VP SEM) (ZEISS IGMA/VF, Jena, Germany, 2012) was used to investigate the morphological structure of fractured surfaces of composites after tensile experiments with an acceleration voltage of 5 kV. A Leica EM SCD 500 high vacuum Sputter Coater (Lecia Microsystems, Prague, Czech Republic, 2012) was employed to gold coat the fracture surfaces with the plasma exposition of 60 s prior to scanning. 

### 2.7. Tensile Test

The tensile test was carried out on dog bone specimens prepared according to EN ISO 527-4 [19], using an MTS EXCEED E45 universal tester (MTS Systems Corporation, Shanghai, China 2016). The specimens were conditioned at 50% relative humidity and 23 °C prior to the testing at the crosshead speed of 1 mm/min. The extensometers were used in the middle of gauge length. The average results of five specimens were recorded for the tensile strength, tensile modulus and elongation at break.

### 2.8. Flexural Test

The bending test was conducted according to EN ISO 14125 [20] on an MTS ECCEED E42 universal tester (MTS Systems Corporation, Shanghai, China) at a speed of 0.5 mm/min. The specimens measured 60 mm × 15 mm × 2 mm with the span length of 40 mm and were preconditioned the environmental method mentioned in the previous test. The flexural strength and modulus were recorded as an average of the five specimens. 

### 2.9. Water Absorption Test

The water absorption test was conducted for all the formulations in accordance with EN ISO 62:2008 [21]. Prior to the test, the samples were conditioned at room temperature with 50% relative humidity after being dried in an oven for 72 h at 40 °C. The specimens were then soaked in two different containers filled with distilled water at the temperatures of 25 °C and 60 °C. The amount of moisture absorption was measured per 24 h for 14 days. $m_{0}$ is the mass of test sample after initial drying and before immersion, and m is the mass of test sample after immersion and final drying. The average mass gain of three test samples for each formulation was calculated and reported. The mass gain (c) is the percentage change in mass relative to the initial mass and is calculated using the Equation (2):

(2) $$c=\frac{m-m_{0}}{m_{0}}\times 100\%$$

## 3. Results

### 3.1. Flammability of Poly(lactic Acid) (PLA), Control and Nano-Hydroxyapatite (nHA)-Filled Composites

The UL-94 results are displayed based on the rate of burning and rating in Table 1. The PLA sample burned continuously until its burn length reached 31 mm and sample drips fell on the chamber bed. For the Control, CN20 and CN30, the UL-94 rating was FH-3, indicating the complete combustion of these composites. The burn rates were recorded 20, 19.6 and 17.7 mm/min, respectively, and no dripping was observed. This demonstrates that increasing volumes of nHA enhanced the flame retardancy. Further addition of nHA (CN40) improved the fire resistivity significantly by providing self-extinguishing performance and no burn rate. This is due to the formation of a more thermally stable char in the respective sample, thereby acting as an effective barrier, shielding the underlying materials from the flame zone and heat. PLA undergoes thermal decomposition via a hydroxyl end-initiated ester interchange process and chain hemolysis creating lactide, oligomers, carbon dioxide, carbon monoxide and acetaldehyde [19], and fillers that can act as flame-retardants are capable of transforming its decomposition pathway to produce char and decrease the formation of combustible products [22,23].

### 3.2. Thermal Studies

**Figure 1:**
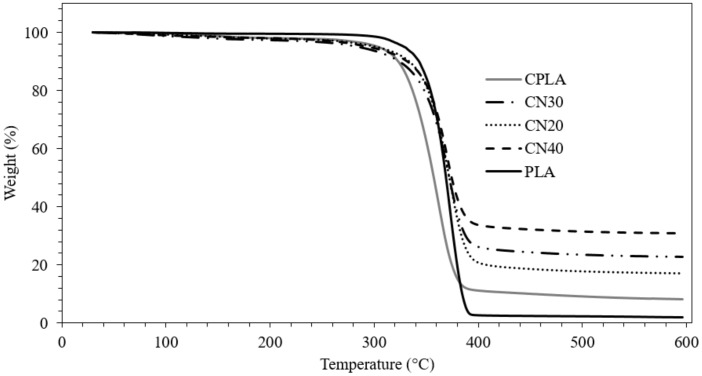
Thermogravimetric analysis (TGA) curves of PLA, control and nanocomposites.

**Figure 2:**
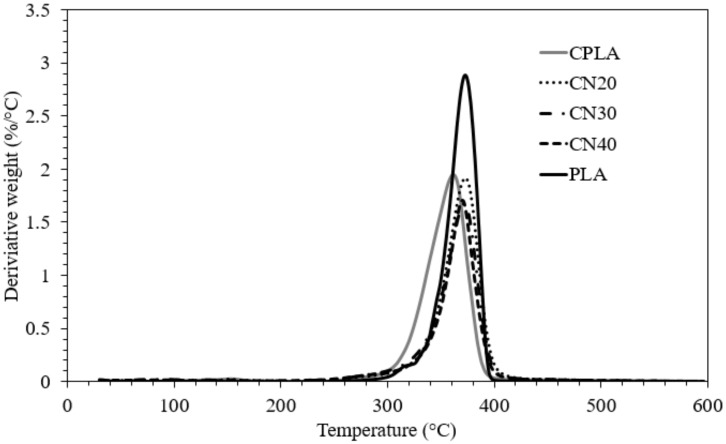
Differential thermogravimetric analysis (DTGA) curves of PLA, control and nanocomposites.

The thermogravimetric analysis (TGA) and differential thermogravimetric analysis (DTGA) curves of the composite samples are shown in Figure 1 and Figure 2. The degradation temperatures, mass residue and mass loss rate of the samples were determined. The TGA curve displayed one decomposition step for all formulations and that the main decomposition took place between 300 °C and 400 °C. It is worth noting that bio-fibres consist of hemicellulose, lignin and cellulose, and their pyrolysis takes place at different temperature ranges of approximately 160–900 °C, 220–315 °C and 315–400 °C for lignin, hemicellulose and cellulose, respectively [24,25]. The temperatures at 5% mass loss (T_5%_) of PLA, CPLA and nanocomposites were approximately 328 °C, 304 °C and 290 °C, and the degradation temperatures (T_d_), obtained from the peak of DTGA curves, were about 373 °C, 361 °C and 372 °C, respectively. This demonstrates that the T_5%_ and T_d_ of CPLA and nHA filled composites are lower than those of PLA, which can be explained by a higher destabilization of PLA in the composites. Amongst the composites, the T_d_ improved with the addition of nHA relative to that of the Control and revealed nearly the same value as neat PLA. It was reported that electrostatic attraction between the polymeric carboxylate group and CA^2+^ of nHA ions affect the interfacial bonding in composites containing nHA [26]. The maximum rates of thermal degradation (Figure 2) were measured 2.88, 1.94, 1.91, 1.7 and 1.67 %/°C for PLA, CPLA, CN20, CN30 and CN40, respectively, suggesting that both the natural fibre and nHA contributed to the reduction in the decomposition rate. The mass residues at 600 °C (Figure 1) were 1.85%, 8.14%, 16.97%, 22.70% and 30.84% for PLA, CPLA, CN20, CN30 and CN40, respectively. The inclusion of natural fibres was observed to improve the mass reside by about 340 % relative to that of neat PLA, and introduction of 40 wt% nHA into the matrix was found to increase the residue by 279% compared to that of CPLA. This enhancement in the increased char residue is in accordance with flammability test, which resulted in the formation of thermally resistive char on the surface of CN40 and subsequent protection of the bulk of the substrate.

### 3.3. Crystallization and Melting Properties

**Figure 3:**
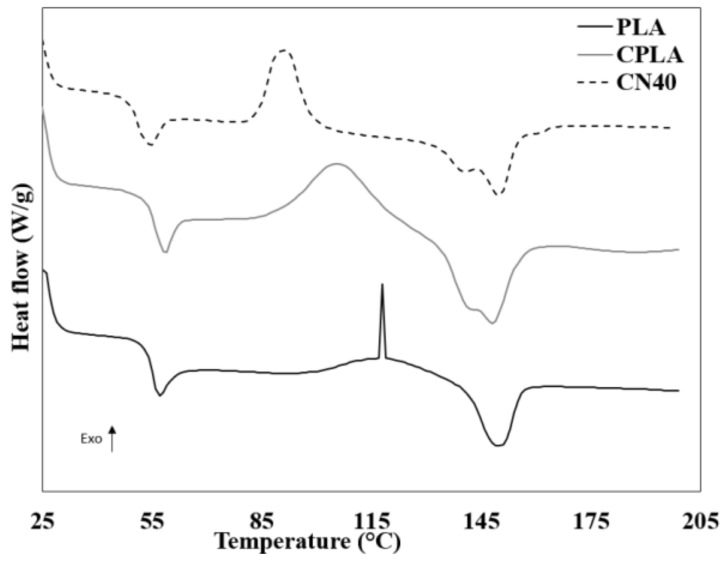
Differential scanning calorimeter (DSC) thermograms of PLA, control (CPLA) and CN40.

The DSC thermograms of CPLA, PLA and CN40 from the heating run are displayed in Figure 3. The glass transition temperature (T_g_), crystallization temperature (T_c_), melting temperature (T_m_), crystallization enthalpy (ΔH_c_), melting enthalpy (ΔH_m_) and degree of crystallinity (X_DSC_) obtained are shown in Table 2. The addition of natural fibres was observed to show an increase in T_g_ compared to that of PLA. This observation displays that an increased T_g_ consequently indicates a change from flexible and soft behaviors to tough and hard properties [27]. The inclusion of nHA slightly decreased the T_g_, indicating improved polymer chain mobility. The crystallinity and T_c_ of CPLA decreased by 12 °C after incorporation of fibres, which implies that the natural fibres hinder the diffusion and migration of molecular chains of PLA to the nucleus surface in CPLA. As expected, the addition of nHA was observed to further reduce the T_c_ by approximately 13%, signifying a faster crystallization of the nanocomposites. This can be ascribed to nHA acting as sites of nucleation, leading to heterogeneous nucleation within the PLA [28]. A similar increase in T_g_ (by approximately. 2 °C) and reduction in T_c_ (by about 14 °C) was obtained after the addition of talc fibre into the PLA [29]. For the last transition, the formation of a smaller second peak is due to the presence of NFs in the PLA which influenced the overall melting behavior of the composites. This suggests the presence of two different types of crystal [30]. Inducing heterogeneous nucleation due to the introduction of nHA can also contribute to the higher T_m_. This is attributed to the creation of less perfect crystals, which would usually melt at greater temperatures than more perfect crystals [15].

### 3.4. Morphological Properties

**Figure 4:**
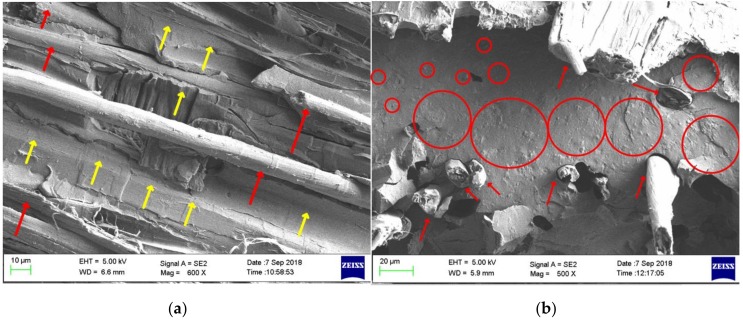

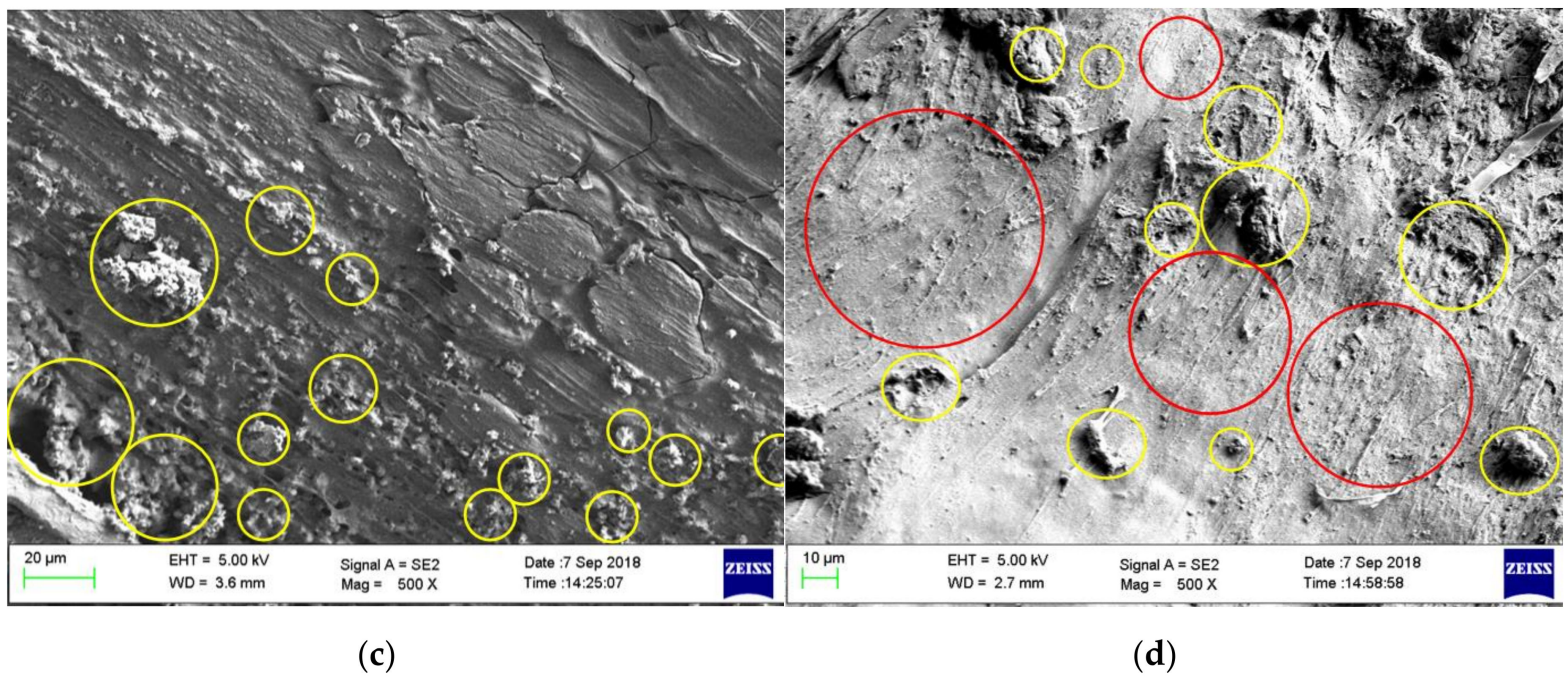
Scanning electron microscope (SEM) micrographs of the fracture surface of tensile tested samples at 500× magnification. (**a**) CPLA, (**b**) CN20, (**c**) CN30 and (**d**) CN40.

The fracture surface of the control and composites containing nHA particles were scanned after tensile tests were performed, as illustrated in Figure 4. As observed from Figure 4a, no fibre pull-out or obvious evidence of poor interfacial bonding in the flax fibre-reinforced PLA can be seen. The red and yellow arrows highlight the flax fibres and PLA matrix in the composite, respectively. The impact of the inclusion of nHA fillers in the composite laminates (CN20, CN30 and CN40) can be observed in their breaking behavior, as shown in Figure 4b–d. A smooth fracture surface was observed in the matrix of CPLA, whereas a rough fracture surface was obtained in the matrix of the nanocomposites. Incorporation of 20 wt% nHA into the matrix was detected to be sufficient to provide even dispersion of particles in the PLA-based matrix, as depicted in the red circles Figure 4b. However, some fibre pull-out and fibre debonding were observed, indicating poor fibre/matrix interfacial adhesion. As the amount of filler increased in the matrix, clear agglomeration was found in the composite laminates ((Figure 4c,d), which is displayed in yellow circles. The agglomerated spots can be points where stress concentration occurs, which can cause premature failure of the composites. It has been stated [28] that well-distributed nHA filler in the PLA matrix can result in improved mechanical performances.

### 3.5. Tensile Properties

**Figure 5:**
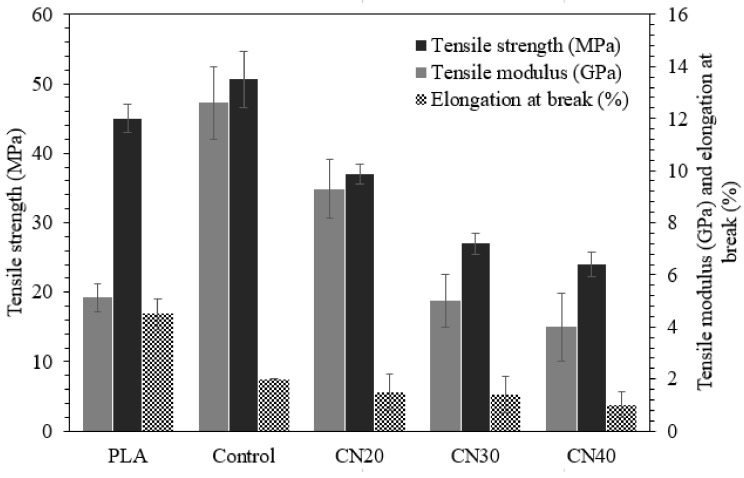
Tensile properties of PLA, control and nHA filled composites.

The tensile properties of the PLA, control and nHA loaded laminates were studied and the results are illustrated in Figure 5. The Young’s moduli of the PLA and Control were 5.1 GPa and 12.6 GPa, the tensile strengths, 45 MPa and 50.7 MPa, and the elongation at break 4.5% and 2%, respectively. As expected, reinforcing the PLA with 6 layers of flax fabric enhanced the tensile strength and modulus by 13% and 146% compared to neat PLA, respectively, which is a significant improvement. This is because tensile performance is primarily fibre-dependent and flax fibre has greater tensile properties than those of neat PLA. Moreover, the observed enhancements in mechanical performance are affected by good interfacial adhesion between matrix and fibre, as demonstrated in Figure 4a, leading to better load transfer between fibre and matrix, and thereby improved mechanical performance. However, after the addition of fabrics, the elongation at break experienced a reduction. As compared to the control, the tensile properties decreased with the inclusion of nHA due to fibre pull-out and fibre debonding. The reduction was substantial upon the addition of 30 wt% and 40 wt% nHA in the matrix, which can be attributed to the low strength of nHA as revealed previously [26,31]. Its low strength can be associated with the elimination of organic compounds during synthesis [15]. Another reason could be the agglomeration of nHA in the matrix, as revealed by the SEM micrographs (Figure 4c,d), which triggers an early failure.

### 3.6. Flexural Properties

**Figure 6:**
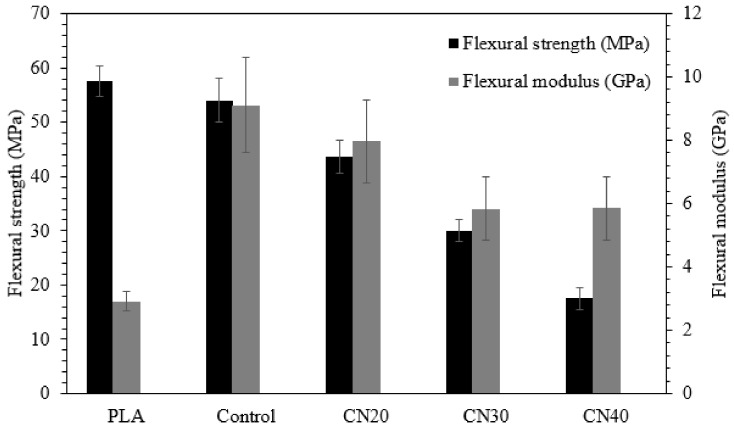
Flexural properties of PLA, control and nHA filled composites.

The flexural performances of the PLA, control and nHA loaded laminates were investigated, and the results are displayed in Figure 6. Neat PLA had a flexural strength and modulus of 57.5 MPa and 2.9 GPa, respectively, whereas the control showed a slightly lower bending strength of 54 MPa and a significantly higher modulus of 9.1 GPa than those of neat PLA, indicating 212% improvement in flexural modulus. As observed in Figure 6, both the flexural strength and the modulus reduced with the increased nHA as compared to those of the control. For instance, for the highest amount of nHA (CN40), the reduction in strength and modulus was 67% and 35%, respectively, relative to those of the control. As mentioned in Section 3.5, the drop can be related to the low strength of nHA, which occurred during its synthesis because of elimination of organic components. The second reason for these reductions is the issue of nHA dispersion in the PLA matrix. Poor dispersion results in agglomeration (Figure 4c,d) which affects the flexural properties as they are matrix dependent. Significant agglomeration can result in unwanted premature failure at the interface of PLA and nHA as well as ineffective load transfer.

### 3.7. Water Absorption Behaviors

**Figure 7:**
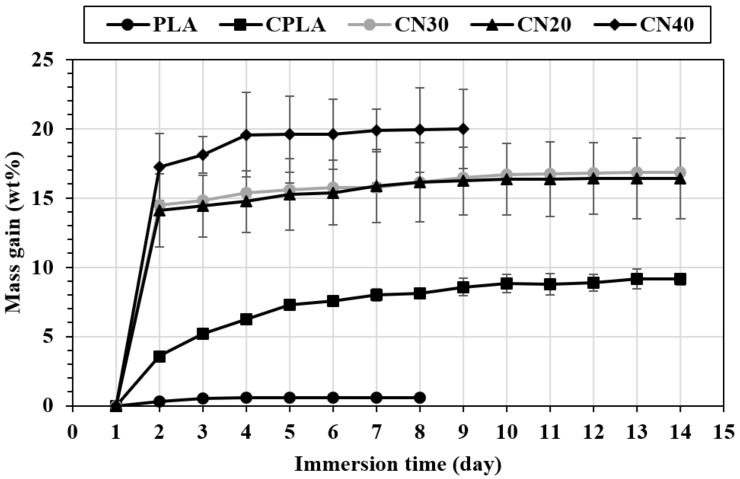
Water absorption behavior of PLA, CPLA and nanocomposites at 23 °C.

**Figure 8:**
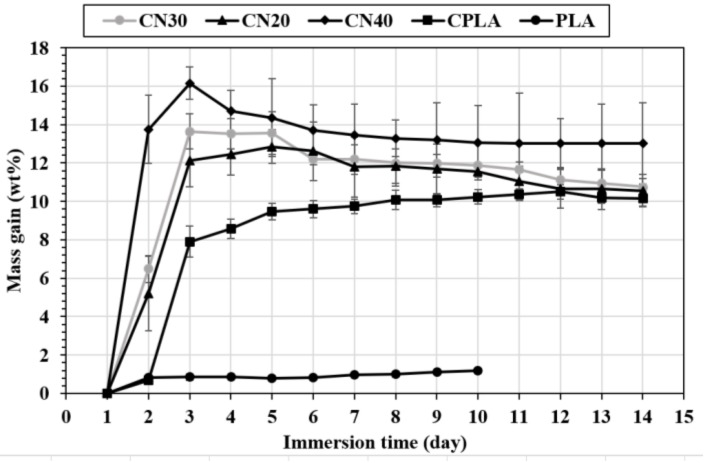
Water absorption behavior of PLA, CPLA and nanocomposites at 60 °C.

The mass gain as a function of the immersion time (day) at 25 °C and 60 °C for the produced composites is displayed in Figure 7 and Figure 8. For PLA at room temperature (Figure 7), the mass gain occurred for two consecutive days and reached approximately 0.6% at the saturation point. The mass remained almost constant for the following days. With the addition of natural fabric into the PLA (CPLA composite), the mass gain increased up to 12 days resulting in a higher saturation value of 8.9%. This is attributed to the hydrophilic nature of flax fibre due to the polar groups e.g., carboxyl and hydroxyl groups. The results obtained are in agreement with another report [32], suggesting that hydrophobic PLA demonstrates a lower tendency to absorb water than lignocellulosic fabrics [33]. For CN20 and CN30, the mass gain continued for 10 days and reached 16.3% and 16.7%, respectively. The saturation time for CN40 was 6 days with the mass gain of 19.8%. This proves that addition of nHA fillers into the matrix increased the water absorption of flax fibre composites, indicating poor interfacial adhesion between the fibres and matrix due to the inclusion of nano fillers, as detected in SEM images (Figure 4), and demonstrated in mechanical analysis. This is because increasing the concentration of nano fillers reduces the amount of PLA in the composite. As a result, less matrix is available to adhere to the natural fibre, thereby exposing the fibres to the environment. Therefore, the natural fibres absorbed more water due to their lack of adhesion to the PLA/nHA matrix. In addition, nHA is a polar (hydrophilic) filler, which further increases the water absorption tendency.

The water absorption behavior of composites immersed in water at 60 °C is displayed in Figure 8. The PLA reached the peak of its mass gain with the value of ca. 0.8% and it became saturated rapidly after about one day. This shows higher amount of mass gain and shorter saturation time as compared to those of PLA at room temperature. For CPLA, the mass gain increased for around 11 days and its value was recorded at 10.4%. These results also show a higher mass gain and shorter saturation relative to those of CPLA soaked at room temperature. After 11 days, the mass of the samples started reducing slightly, which was due to the separation of tiny PLA pieces as a result of disintegration of the water-soluble materials [32]. The CN20, CN30 and CN40 samples saturated rapidly, after 4, 2 and 2 days, when compared to the saturation time obtained at room temperature, respectively. The mass gain was higher for the samples with greater concentration of nHA. These composites experienced a continuous mass loss after the peak of mass gain due to peeling and degradation of the nHA/PLA samples. This could be due to the poor interfacial adhesion between the nHA/PLA matrix and the flax fibre, thereby creating a higher surface area for degradation of the matrix material. It is worth highlighting that more cracks were visibly be observed on the surface of composites reinforced with flax fibre than neat PLA, and the cracks were more obvious for composites containing higher concentrations of nHA.

## 4. Conclusions

This research studied environmentally friendly biocomposites which were prepared using hot compression with the incorporation of fully bio-sourced constituents (i.e., bio-filler, NF and bio-thermoplastic). The study demonstrates the flame resistivity and thermal improvement of nHA as an additive by preventing flame development in the composite specimen (CN40) and reducing the burn rate as obtained via the UL-94 test study. It should also be noted that the mass residue was enhanced by 279%, thermal decomposition temperature was improved slightly, and the mass loss rate was reduced by approximately 14% upon the addition of the nanofillers determined by the initial TGA investigation. The efficiencies were more pronounced in terms of flame retardancy and thermal resistivity at higher concentrations of nHA particles. From the DSC analysis, it was found that the degree of crystallinity reduced in CPLA when compared to that of neat PLA, and with the presence of nHA additives in the composite, X_DSC_ experienced an increase as compared to its value of CPLA. The nHA particles, while imparting good thermal resistivity to the composite laminates, induced lower mechanical properties at higher concentrations. This reduction could be attributed to the agglomeration of the nHA particles within the matrix, in particular at higher concentrations, as well as increased fibre pull-out/debonding upon nHA inclusion, based on morphological observations. The water absorption increased with the addition of NF as well as nHA particles at both room temperature and 60 °C, which is ascribed to the poor adhesion at the interface between nHA/PLA matrix and flax fibres, as validated in the SEM micrograph, thereby enabling easier moisture absorption of NFs in the composites. This is because there is less matrix available to coat the natural fibres, which results in easy exposure to the environment. Furthermore, water absorption tendency increases in the presence of inorganic fillers such as nHA. At higher temperature, the saturation time was shortened remarkably for all the composites. In addition, the matrixof composite specimens started to disintegrate throughout the test due to the hydrolytic degradation. Therefore, there must be a compromise between the flame retardancy/thermal resistivity and mechanical/moisture absorption behavior for these types of biocomposites.

## Figures and Tables

**Table 1:** UL-94 results of poly(lactic acid) (PLA), Control and nano-hydroxyapatite (nHA) filled composite laminates.

Sample	Burn Rate (mm/min)	UL 94 Rating
PLA	NA	FH-2-31 mm
CPLA	20	FH-3
CN20	19.6	FH-3
CN30	17.7	FH-3
CN40	NA	FH-1

**Table 2:** The T_g_, T_c_, T_m_, ΔH_c_, ΔH_m_ and X_DSC_ obtained from DSC test.

Samples	T_g_ (°C)	T_c_ (°C)	T_m_ (°C)	ΔH_c_ (J/g)	ΔH_m_ (J/g)	X_DSC_ (%)
PLA	57	118	150	6.02	9.04	4.95
CPLA	59	106	148	17.47	18.41	1.54
CN40	56	92	151	13.83	15.04	1.98
